# Bone health and body composition in transgender adults before gender-affirming hormonal therapy: data from the COMET study

**DOI:** 10.1007/s40618-023-02156-7

**Published:** 2023-07-14

**Authors:** C. Ceolin, A. Scala, M. Dall’Agnol, C. Ziliotto, A. Delbarba, P. Facondo, A. Citron, B. Vescovi, S. Pasqualini, S. Giannini, V. Camozzi, C. Cappelli, A. Bertocco, M. De Rui, A. Coin, G. Sergi, A. Ferlin, A. Garolla, the Gender Incongruence Interdisciplinary Group (GIIG), Andrea Garolla, Anna Aprile, Bruno Azzena, Camillo Barbisan, Valentina Camozzi, Elena Campello, Cattelan Annamaria, Chiara Ceolin, Fabrizio Moro, Giorgio Conti, Angela Favaro, Alberto Ferlin, Francesco Francini, Michela Gatta, Marta Ghisi, Sandro Giannini, Laura Guazzarotti, Massimo Iafrate, Paolo Meneguzzo, Marina Miscioscia, Giancarlo Ottaviano, Carlo Saccardi, Lolita Sasset, Alberto Scala, Rossana Schiavo, Giuseppe Sergi, Paolo Simioni, Benedetta Tascini, Francesca Venturini, Fabrizio Vianello

**Affiliations:** 1 Geriatrics Division, Department of Medicine (DIMED), University of Padua, Padua, Italy; 2 Unit of Andrology and Reproductive Medicine, Department of Medicine (DIMED), University of Padua, Padua, Italy; 3 Unit of Endocrinology and Metabolism, University of Brescia and ASST Spedali Civili, Brescia, Italy; 4 Clinica Medica 1, Department of Medicine, University of Padua, Padua, Italy; 5 Endocrinology Unit, Department of Medicine (DIMED), University of Padua, Padua, Italy; 6 Regional Reference Center for Gender Incongruence, Padua, Veneto Region Italy

**Keywords:** Bone metabolism, Body composition, Transgender, Lean mass, Fat mass

## Abstract

**Purpose:**

Preliminary data suggested that bone mineral density (BMD) in transgender adults before initiating gender-affirming hormone therapy (GAHT) is lower when compared to cisgender controls. In this study, we analyzed bone metabolism in a sample of transgender adults before GAHT, and its possible correlation with biochemical profile, body composition and lifestyle habits (i.e., tobacco smoke and physical activity).

**Methods:**

Medical data, smoking habits, phospho-calcic and hormonal blood tests and densitometric parameters were collected in a sample of 125 transgender adults, 78 Assigned Females At Birth (AFAB) and 47 Assigned Males At Birth (AMAB) before GAHT initiation and 146 cisgender controls (57 females and 89 males) matched by sex assigned at birth and age. 55 transgender and 46 cisgender controls also underwent a complete body composition evaluation and assessment of physical activity using the International Physical Activity Questionnaire (IPAQ).

**Results:**

14.3% of transgender and 6.2% of cisgender sample, respectively, had z-score values < -2 (*p* = 0.04). We observed only lower vitamin D values in transgender sample regarding biochemical/hormonal profile. AFAB transgender people had more total fat mass, while AMAB transgender individuals had reduced total lean mass as compared to cisgender people (53.94 ± 7.74 vs 58.38 ± 6.91, *p* < 0.05). AFAB transgender adults were more likely to be active smokers and tend to spend more time indoor. Fat Mass Index (FMI) was correlated with lumbar and femur BMD both in transgender individuals, while no correlations were found between lean mass parameters and BMD in AMAB transgender people.

**Conclusions:**

Body composition and lifestyle factors could contribute to low BMD in transgender adults before GAHT.

**Supplementary Information:**

The online version contains supplementary material available at 10.1007/s40618-023-02156-7.

## Introduction

*Transgender* is an umbrella term that refers to people whose gender identity, transiently or persistently, differs from the sex assigned at birth. Transgender people may describe their gender as male or female, or have a non-binary identity. People whose gender identity corresponds to their sex assigned at birth are referred to as *cisgender* [1]. Terms such as Assigned Females At Birth (AFAB) and Assigned Males At Birth (AMAB) are used in the scientific literature to refer to transgender people [2]. Trans women and trans men are widely used terms, too. Gender dysphoria (GD) is defined as a condition of psychological distress associated with gender incongruence [3].

Although not all transgender people experience GD or require medical intervention, some of these individuals may benefit from tailored medical interventions to change their primary and secondary sex characteristics. Gender-affirming care requires a multidisciplinary management that includes mental health care, gender-affirming hormone therapy (GAHT), and/or gender-affirming surgery (GAS) [1, 4]. In GAHT, testosterone and a combination of estrogen and anti-androgen drugs (e.g., cyproterone acetate and spironolactone) are commonly used in AFAB and AMAB individuals, respectively. During puberty, the development of secondary sex characteristics can worsen GD in gender-variant children. Gonadotropin-releasing hormone analogues (GnRHa) can reversibly block puberty, until sufficient maturity is acquired to discuss GAHT and other irreversible treatments [1, 4]. Given the importance of sex hormones on bone physiology, much attention has been paid to the effect of GAHT on bone mineralization over the years [5–10], while only few authors analyzed bone conditions before initiating GAHT [5, 11–13]. Lower bone mineral density (BMD) values were observed in AMAB trans people when compared to cisgender men, however, the reasons have not yet been fully clarified [11]. Given the absence of significant abnormalities in the hormonal profile of the transgender patients, Van Caenegem et al. [11, 13] hypothesized that lifestyle factors, such as use of alcohol and tobacco, social isolation and low physical activity, could be important risk factors, with direct and indirect influence on bone development during puberty.

In this study, we analyzed bone metabolism in a sample of transgender adults before the initiation of GAHT. Furthermore, we evaluated the possible correlations between bone density and biochemical profile, body composition and lifestyle habits (i.e., tobacco smoke and physical activity).

## Materials and methods

### Study population

The “Body COmposition and bone MEtabolism in Transgender adults” (COMET) study is a retrospective study conducted on 271 adults: 125 transgender individuals (78 AFAB, 47 AMAB) and 146 cisgender controls (57 female, 89 male). We included transgender people who were evaluated at the Unit of Andrology and Reproductive Medicine of the University Hospital of Padua (Italy) and the Unit of Endocrinology of the Hospital of Brescia (Italy), between May 2018 and March 2023. Inclusion criteria were: diagnosis of GD; evaluation before GAHT and/or GAS; 20 to 50 years of age; pre-menopausal female at birth participants; Body Mass Index (BMI) 19–35 kg/m^2^. Exclusion criteria were: chronic use of drugs affecting bone metabolism (e.g., glucocorticoids, thyroxine, immunosuppressants, NSAIDs, PPIs, diuretics, vitamin D, and calcium); history of hyperparathyroidism; use of oral contraceptive pills.

The control population was composed of 146 cisgender individuals matched by age (± 2 years, median 1 year) and sex assigned at birth. Cisgender volunteers were recruited at the University Hospital of Padova.

The study protocol was approved by the local Ethics Committee (Comitato Etico per la Sperimentazione Clinica della Provincia di Padova, number 0025087) and complies with the guidelines of the Declaration of Helsinki. Each individual gave their written consent to participate in the study.

### Data collection

For each participant, the following information were collected by trained physicians through personal interview, physical examination and medical records.

*Patient characteristics:* Physiological, clinical and pharmacological data were collected for each participant, including tobacco use (active or former smoker; number of cigarettes/day).

*Anthropometry*. Weight and height measurements were carried out with participants wearing light indoor clothes and no shoes. Waist circumference was measured midway between the lowest rib and the iliac crest with participants standing [14]. Hip and calf circumference was measured around the widest portion of the buttocks and at the maximum circumference in the middle of the calf length, respectively [15]. Finally, mid–upper arm circumference (MUAC) was measured in the right upper arm at the midpoint between the tip of the shoulder and the tip of the olecranon process [16].

*Laboratory data.* Blood samples were collected fasting in the early morning to perform the following tests: estradiol, total testosterone, luteinizing hormone (LH), follicle-stimulating hormone (FSH), serum calcium and phosphate, parathyroid hormone (PTH), 25-hydroxy-vitamin D (25-OH-D). Test analysis was performed following the same procedures at the Laboratory Medicine Unit of the University Hospital of Padova and the Hospital of Brescia.

*Physical Performance measures.* Upper limbs strength was evaluated with handgrip strength test. Measurement was made with DynEx electronic hand dynamometers (Ohio, USA) by trained medical personnel. Three trials were carried out for each hand, and grip strength was calculated as the mean of the maximum performance at the dominant and no-dominant hand.

*Evaluation of bone density and body composition*. BMD was assessed using Dual Energy X-ray Absorptiometry (DXA) using fan-beam technology (Hologic QDR 4500 W, Inc.) at proximal femur (femoral neck and/or total hip) and lumbar spine in each patient. Since the age of our patients is under 50 years, Z-scores was used for all the analyses in accordance with the World Health Organization (WHO) recommendation [17]. People were considered having low BMD if total hip, femoral neck and/or lumbar spine Z-score was ≤ – 2 [18]. Total body DXA examination was carried out to measure Fat-free mass (FFM), Fat mass (FM), and Appendicular Skeletal Muscle Mass (ASMM). The Indices of Fat Mass (FM-Index) and Appendicular Skeletal Muscle Mass (ASMM-Index) were calculated dividing the FM and ASMM by the height in squared meters.

*Questionnaires:* As part of a multidimensional assessment, patients were asked to fill in the following questionnaire:

- International Physical Activity Questionnaire (IPAQ): it investigates physical activity levels during the last 7 days. IPAQ results were expressed as Metabolic Equivalent of Task (MET) and calculated by multiplying the MET assigned to it (vigorous—8 MET, moderate—4 MET and walking—3.3 MET) by the number of days it was performed during a week, where MET corresponds to O_2_ consumption during the rest and equals 3.5 mL O_2_/kg of the body mass per minute. The total MET value was then computed by summing up the respective MET values for all activities that were carried out in bouts longer than 10 min in duration [19].

### Statistical analysis

Categorical variables are expressed as counts and percentages and continuous quantitative variables as mean ± standard deviation or median (interquartile range). Normal distribution of continuous variables was verified with a Shapiro–Wilk test. Variables were compared by sex assigned at birth by Mann–Whitney and Kruskal–Wallis tests for quantitative variables, and by Chi-square test for categorical variables. Correlations were performed using Pearson’s correlation coefficient (r) or Spearman’s rank correlation coefficient (r_s_) when variables were not normally distributed. In all analyses, significance was assumed if *p* ≤ 0.05. Analysis was performed with statistical software IBM SPSS version 29 (IBM Corp., Armonk, NY).

## Results

### Baseline data

Sample characteristics, densitometric values and phospho-calcic metabolism parameters at baseline are displayed in Table 1. One hundred and twenty five transgender adults were matched for age and sex assigned at birth with 146 cisgender controls. The transgender group consisted of 78 AFAB (62.4%) and 47 AMAB (37.6%), who were compared with 57 female and 89 male cisgender adults. No difference in height, weight, and BMI was found. None of the individuals had significant comorbidities, nor took medications regularly. We observed an increased number of active smokers in transgender individuals (*p* < 0.05). No significant difference in the hormonal profile was found between the transgender and the cisgender group (please see Supplementary table S1).

**Table 1:** Characteristics at baseline in transgender and cisgender people

	Transgender (*n* = 78) AFAB	Cisgender (*n* = 57) Females	Transgender (*n* = 47) AMAB	Cisgender (*n* = 89) Males
Age [years]	26.09 ± 7.42	27.66 ± 5.81	26.89 ± 8.72	24.89 ± 5.36
Active smokers [%]	36 (46.2%)	20 (35%)^*^	12 (25.5%)	10 (11.25%)^*^
BMI [kg/m^2^]	23.99 ± 6.67	22.62 ± 3.12	22.76 ± 4.11	23.67 ± 4.02
Densitometric parameters				
z-score lumbar	− 0.27 ± 1.12	− 0.20 ± 1.76	− 0.99 ± 1.26	− 0.08 ± 1.22^***^
z-score femur	− 0.18 ± 1.08	0.04 ± 0.74	− 0.66 ± 0.83	0.08 ± 0.81^***^
z-score femur neck	− 0.32 ± 1.07	− 0.07 ± 0.80	− 0.71 ± 0.80	0.08 ± 0.93^***^
Biochemical parameters				
25-OHD [nmol/L]	52.50 (36.15;66.25)	80.00^***^ **(38.00;112.00)**	52.38 (34.81;71.87)	74.50^***^ **(61.75;87.25)**
Calcium [mg/dL]	9.38 (9.10;9.70)	9.60 (9.20;10.24)	9.83 (9.73;10.00)	9.74 (9.36;9.96)
Phosphate [mg/dL]	3.50 (3.01;4.10)	3.19 (2.88;3.38)	3.30 (2.95;3.63)	3.13 (2.76;3.61)
PTH [ng/L]	38.00 (24.00;56.00)	30.00 (22.00;47.50)	32.90 (17.82;49.50)	30.25 (20.75;52.00)

Values are expressed as mean ± standard deviation or as median (interquartile range) or as count (percentages) as appropriate

The *p *values < 0.05 are shown in bold

AFAB assigned female at birth, AMAB assigned male at birth, *BMI* body mass index, *25-OHD* 25-hydroxy vitamin D, *PTH* parathyroid hormone

*p < 0.05; **p < 0.01; ***p < 0.001

#### Densitometric values

**Fig. 1:**
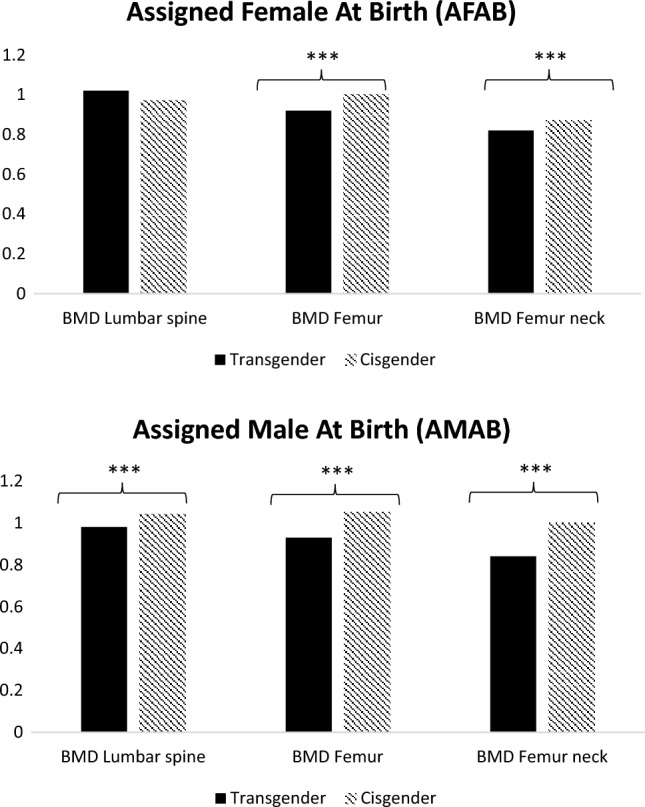
Distribution of BMD values in transgender and cisgender people. Lumbar spine, total hip, and femoral neck sites were considered. ***p < 0.001, *AFAB* assigned female at birth, *AMAB* Assigned Male At Birth, *BMD* bone mass density

Considering densitometric values, 14.3% of transgender and 6.2% of cisgender sample, respectively, had z-score values < -2 (*p* = 0.04, data not shown). In particular, AMAB transgender adults had lower z-scores in all sites analyzed (lumbar, total femur and femur neck) with consistently lower BMD values (Fig. 1). In addition, in AFAB transgender people, we observed lower BMD values, especially at femur site (*p* < 0.001, Fig. 1), with no significant difference of z-score values.

#### Biochemical results

In regard to phospho-calcic metabolism, vitamin D levels were significantly lower in the transgender population (52.00 vs 80.00 nmol/L in AFAB and 52.38 vs 74.50 nmol/L in AMAB, respectively), while the values of calcium, phosphor, and PTH were similar to controls.

**Fig. 2:**
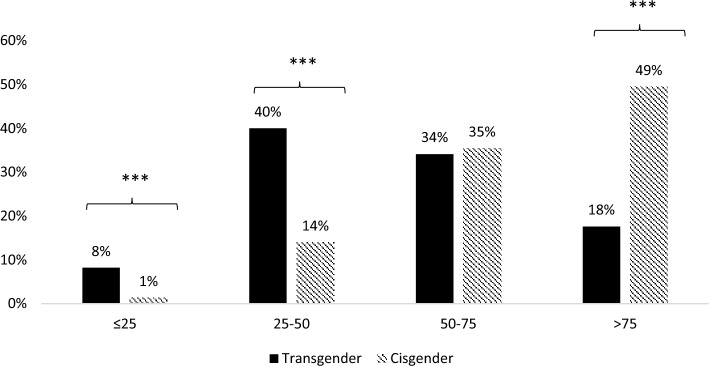
Serum 25-OHD values in transgender and cisgender population and cutoff as: ≤ 25; 25–50; 50–75; > 75 nmol/L. ***p < 0.001

There was a great percentage of transgender people with 25-hydroxy-colecalciferol (25-OHD) levels < 25 nmol/L (8% vs 1%, *p* < 0.001) and between 25 and 50 nmol/L when compared to cisgender controls. On the contrary, a great number of cisgender adults had levels > 75 nmol/L (49% vs 18%, *p* < 0.001) (Fig. 2).

### Body composition

Out of 271 individuals studied, 55 transgender (29 AFAB and 26 AMAB) and 46 cisgender people (26 AFAB and 20 AMAB) underwent a complete body composition evaluation.

**Fig. 3:**
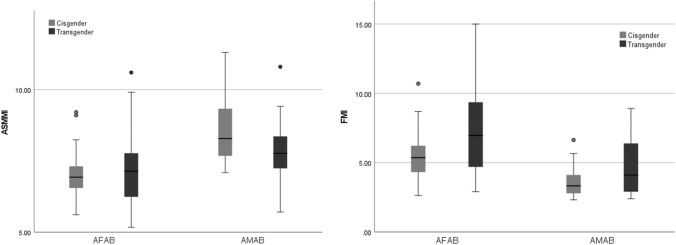
Distribution of ASMMI and FMI in transgender and cisgender people

Table 2 shows the body composition parameters of transgender and cisgender population. Hips and arm circumferences were larger in AFAB transgender individuals (96.05 ± 12.78 vs 88.70 ± 10.31 and 28.29 ± 4.94 vs 26.06 ± 2.42, *p* < 0.05, respectively), while no significant differences were observed in AMAB population. AFAB transgender people had more total fat mass and FMI (21.24 ± 10.29 vs 14.90 ± 5.53, *p* < 0.001 and 7.58 ± 3.50 vs 5.53 ± 1.62, *p* < 0.01, respectively, Fig. 3), while lean mass parameters were similar to cisgender peers. On the contrary, in AMAB transgender people we observed a reduction of muscle mass, as well as upper limbs strength. In fact, both total lean mass and ASMMI were reduced when compared to male cisgender adults (53.94 ± 7.74 vs 58.38 ± 6.91 and 7.80 ± 1.04 vs 8.54 ± 1.16, *p* < 0.05, respectively, Fig. 3). Furthermore, bone mineral content (BMC) and handgrip max test values were significantly lower in transgender AMAB individuals (handgrip maximum strength: 35.46 ± 9.98 vs 45.32 ± 11.66, *p* < 0.01).

**Table 2:** Body composition parameters at baseline in transgender and cisgender people

	Transgender (*n* = 29) AFAB	Cisgender (*n* = 26) Females	Transgender (*n* = 26) AMAB	Cisgender (*n* = 20) Males
Age [years]	23.59 ± 5.61	24.00 ± 4.47	25.73 ± 7.19	23.35 ± 4.52
Active smoke [%]	12 (41.4%)	5 (29.4%)^*^	9 (34%)	3 (20%)^*^
Number cigarettes/die	3.41 ± 4.57	3.10 ± 5.30	1.27 ± 2.89	3.01 ± 1.15
BMI [kg/m^2^]	25.46 ± 9.04	21.75 ± 2.36	22.46 ± 3.52	22.21 ± 2.04
Circumferences [cm]				
Waist	80.06 ± 12.48	78.10 ± 8.73	82.25 ± 9.68	83.47 ± 8.12
Hips	96.05 ± 12.78	88.70 ± 10.31^*^	91.90 ± 7.66	91.35 ± 7.82
Calf	36.29 ± 4.80	35.84 ± 2.80	35.96 ± 3.27	36.95 ± 3.41
Arm	28.29 ± 4.94	26.06 ± 2.42^*^	27.42 ± 2.59	28.60 ± 3.29
Body composition parameters				
Total body mass [kg]	67.57 ± 19.92	62.74 ± 9.18	68.54 ± 13.70	72.38 ± 8.44
Total fat mass [kg]	21.24 ± 10.29	14.90 ± 5.53^***^	14.27 ± 5.92	11.61 ± 3.64
Total lean mass [kg]	44.99 ± 7.58	45.40 ± 5.90	53.94 ± 7.74	58.38 ± 6.91^*^
BMC [g]	2.23 ± 0.32	2.26 ± 0.29	2.44 ± 0.24	2.66 ± 0.42^*^
ASMMI-Index [kg/m^2^]	7.06 ± 1.25	7.07 ± 0.90	7.80 ± 1.04	8.54 ± 1.16^*^
FM-Index [kg/m^2^]	7.58 ± 3.50	5.53 ± 1.62^**^	4.68 ± 2.05	3.63 ± 1.13^*^
Handgrip max test [kg_f_]	30.22 ± 5.61	30.80 ± 7.12	35.46 ± 9.98	45.32 ± 11.66^**^
Physical activity [METs]				
At work	2772.00 (0.00;4597.00)	1095.00 (1095.00;2160.00)	0.00 (0.00;315.00)	75.00 (0.00;1095.00)
Outdoor	525.00 (210.00;1192.00)	1170.00 (900.00;1170.00)	1125.00 (180.00;1657.50)	1215.00 (690.00;2707.00)
Indoor	1080.00 (300.00;1210.00)	240.00^*^ **(240.00;720.00)**	630.00 (75.00;1210.00)	480.00 (240.00;1140.00)

Values are expressed as mean ± standard deviation or as median (interquartile range) or as count (percentages) as appropriate

The *p *values < 0.05 are shown in bold

*AFAB* assigned female at birth, *AMAB* assigned male at birth, *BMI* body mass index, *BMC* bone mineral content, *ASMMI* appendicular skeletal muscle mass index, *FM* fat mass index, *MET* metabolic equivalent of task

*p < 0.05; **p < 0.01; ***p < 0.001

At last, physical activity evaluation revealed that AFAB transgender adults spent more time than cisgender inside their house (*p* = 0.04) and tended to less time outside.

### Correlations

Pearson’s correlations between lumbar, total and neck femoral BMD and covariates in transgender people are reported in Table 3. In AFAB adults, total femur and femur neck BMD were positively correlated with BMI (*r* = 0.38, *p* < 0.01 and *r* = 0.28, *p* < 0.05, respectively), ASMMI (*r* = 0.45 and *r* = 0.41, *p* < 0.05, respectively), FMI (*r* = 0.62, *p* < 0.01, respectively) and handgrip strength; a negative correlation was found with age (*r* = − 0.29 and r = − 0.37, *p* < 0.05, respectively). Lumbar spine BMD was associated to BMI, ASMMI and FMI values. In AMAB people, lumbar spine and total femur BMD were positively correlated with FMI (*r* = 0.39 and *r* = 0.51, *p* < 0.05, respectively).

**Table 3:** Pearson’s correlations between bone mineral density (BMD) and covariates in transgender people

	AFAB			AMAB		
	Lumbar spine BMD	Total femur BMD	Femur neck BMD	Lumbar BMD	Total femur BMD	Femur neck BMD
*Age*	− 0.23	– 0.29*	– 0.37*	0.14	– 0.03	– 0.20
*Smoke habit*	0.01	– 0.02	0.06	0.08	0.22	0.25
*BMI*	0.28*	0.38**	0.28*	0.24	0.22	0.23
*ASMMI*	0.53*	0.45*	0.41*	0.14	– 0.08	0.01
*FMI*	0.39*	0.62**	0.62**	0.39*	0.51*	0.38
*HGM*	0.32	0.45*	0.54*	– 0.04	– 0.14	– 0.02
*Vitamin D*	0.05	0.06	0.07	0.09	0.07	0.02
*Estradiol*	0.07	– 0.09	– 0.11	0.31	0.30	0.35
*Total testosterone*	– 0.03	– 0.11	0.15	0.08	0.12	0.22
*LH*	– 0.10	0.12	– 0.11	– 0.23	– 0.10	– 0.11
*FSH*	0.13	0.12	0.10	0.16	0.05	– 0.04
*MET—t work*	0.04	0.28	0.37	0.44*	0.07	0.20
*MET—Indoor*	0.01	0.24	0.29	0.20	– 0.08	– 0.07
*MET—Outdoor*	– 0.01	0.06	0.18	0.29	0.26	0.10

The *p *values < 0.05 are shown in bold

*AFAB* assigned female at birth, *AMAB* assigned male at birth, *BMI* body mass index, *BMD* Bone Mineral Density, *ASMMI* appendicular skeletal muscle mass index, *FMI* fat mass index, *HGM* handgrip max strength, *LH* luteinizing hormone, *FSH* follicle-stimulating hormone

*p < 0.05; **p < 0.01; ***p < 0.001

## Discussion

Our study demonstrated that transgender adults have reduced bone mass before the initiation of GAHT, when compared to cisgender controls. Also, despite similar BMI and total body mass, we observed that AFAB individuals had more fat mass and AMAB people had less lean mass in comparison to cisgender controls, with potential bone health implications.

Only few authors investigated bone metabolism in transgender people before GAHT. In line with our findings, previous studies observed lower BMD values both at lumbar spine and femoral sites in AMAB transgender individuals [3, 7, 11, 12, 20]. In AFAB individuals, bone mineralization and geometry were described as similar [13, 21] or even slightly better [12] than cisgender controls. We found significantly lower BMD at the hip site in AFAB transgender adults, which, however, was not accompanied by a reduction in z-score values; no difference was found at lumbar site.

In line with the scientific literature [5, 11, 22], we found no difference in hormonal and mineral metabolism between transgender people and controls, except for vitamin D. Both AFAB and AMAB transgender people had reduced levels of vitamin D when compared to controls. In particular, a great percentage of transgender people (82%) had less than 75 nmol/L of vitamin D. According to the Endocrine Society guidelines, values of 25-OHD between 51 and 75 nmol/L identify a vitamin D insufficiency and values < 50 nmol/L a deficiency [23]. Anyway, the term hypovitaminosis D is widely used to refer to both conditions. Vitamin D plays a key role in bone metabolism: it increases calcium reabsorption in kidney and intestine, and reduces PTH secretion in parathyroid glands, thus inhibiting calcium reabsorption from bone [24]. Most studies have found a greater prevalence of hypovitaminosis D in the transgender population, but data on the effects of vitamin D in this population are still lacking [25]. A multicentric observational study [7] found that vitamin D supplementation was associated with a greater increase of lumbar BMD in transgender adults during GAHT.

Nonetheless, we found no association between vitamin D values and BMD, in contrast with previously reported data [26–28]. This discrepancy with previous studies could be due to: a) young age of participants in the COMET study, which could diminish the cumulative effect of hypovitaminosis D on bone density over lifetime and b) the average level of vitamin D in our sample falls under the category of insufficiency – not deficiency.

In addition, lifestyle factors could play an important role in vitamin D metabolism. In fact, physical activity promotes the absorption of some micronutrients, including vitamin D. Also, sun exposition during outdoors activities is essential for the biosynthesis of vitamin D. Interestingly, previous studies highlighted that transgender people tend to participate less in sports and physical activities [29].

Finally, considering the high percentage of active smokers in our transgender population, tobacco could contribute to reduce serum 25-OHD [30]. Although the mechanism at the base of smoke-induced hypovitaminosis D has not been fully understood, tobacco may alter hepatic metabolism of vitamin D [31, 32].

Surprisingly, we did not observe significant correlations between active smoking habits or physical activity and BMD, despite the effect on bone health of these conditions is well known [31]. Interestingly, cigarettes can interfere in the production, metabolism, and binding of estradiol [31]. In transgender people, higher levels of estradiol during GAHT were associated with better BMD values [5, 7, 10]. We suspect that the deleterious effects of tobacco on bone might not be visible in this population, yet. In fact, the smoke-induced bone damage is specifically influenced by dose and duration [33]. Relatively young age and low numerosity of our sample might have masked the deleterious effects of smoke on bone. Furthermore, active smoking was more prevalent solely in the AFAB group, in which bone density was similar to cisgender controls.

Anyway, smoke remains an important risk factor for bone health and cessation of tobacco use should be strongly recommended in transgender individuals [31]. In regards to physical activity, the IPAQ questionnaire did not found a marked difference between cisgender and transgender people, but there was a tendency in AFAB transgender individuals to spend more time indoors and practice less outdoor activities. Although our findings did not reach statistical significance, larger studies confirmed a reduction in physical activity in transgender youth. Data from the Minnesota Student Survey, 2016 revealed that a large share of transgender adolescents did not participate in sports (73.8% vs 44,6%) or were physically inactive (23.9% vs 10.2%) [29]. An online survey found similar results in Spain [34].

Therefore, a reduction in physical activity could partially explain the different body composition and bone density found in our transgender sample. In fact, mechanical load induced by skeletal muscles is essential for bone development during puberty [35].

Hormone therapy has the ability to modify body composition and adipose tissue distribution in order to achieve a more masculine or feminine body appearance. In fact, testosterone increases muscle mass and strength, reduces fat mass, and produces an androgenic (i.e., abdominal) body fat distribution, while estrogen has opposite effects [1].

Our study showed that AFAB transgender people had more fat mass and AMAB individuals had lower lean mass, despite similar BMI and total body mass. In transwomen, muscle mass reduction was associated with lower handgrip strength. In line with our findings, Van Caenegem et al. [11] reported lower BMD, lean mass, and strength in this population. Instead, AFAB individuals had similar values of lean and fat mass in comparison to cisgender women [20–22]. Exploring the correlation between body composition and bone mineral density, we observed different results according to sex assigned at birth: in AFAB transgender adults, ASMMI had a strong correlation with lumbar BMD, while FMI was positively correlated to femur BMD. In the AMAB group, we only found an association between FMI and lumbar and femur BMD, in contrast to previous studies [20]. These results lead to reflect on the role of body composition on bone health: it is possible that a reduction of lean mass in AMAB transgender adults is partly responsible for the lower BMD and z-score values observed at all sites. This could have important implications especially after starting hormonal therapy, since estrogen maintains bone density, even though it reduces lean mass in favor of body fat [36]. A similar phenomenon has been observed in the cisgender population. In fact, women have a greater bone mass relative to total muscle mass and body weight, even after adjustment for fat mass. Evidence suggests that estrogen increases bone sensitivity to mechanical loading. As a result, BMD values are similar in men and women, despite the latter have less lean mass [35].

The role of muscle mass on bone development, especially during puberty, is well known in the cisgender population [37, 38]. During adolescence, mechanical loading of skeletal muscles contributes to the achievement of peak bone mass [39–44]. To reinforce these data, studies conducted on young people revealed a positive correlation between lean mass and BMD, especially in males [42, 45, 46]. On the contrary, the effect of adipose tissue is more debatable. In fact, an excess of adipose tissue seems to negatively influence bone development. On the other hand, adipocytes release adipokines, cytokines, and estrogens which stimulate bone deposition [41–43, 47]. Adipose tissue can, therefore, produce positive effects on bone density, as observed in young people, especially in female individuals [46]. Given these considerations, we hypothesize that adipose tissue may be a protective factor for bone mass in AMAB transgender people, partially compensating for lower levels of muscle mass. It is possible that AMAB individuals experience an inadequate peak bone mass during adolescence, partially due to the differences in body composition and lifestyle factors observed in our research.

### Limitations

Limitations of our study included small sample size. Due to the rarity of this condition, the number of participants limited the statistical power of analyses. Furthermore, markers of bone formation and resorption, and 24-h urine calcium values were not available. In regards to vitamin D, seasonality was ruled out as all participants were recruited throughout the entire year. Finally, we did not perform a dietary investigation in this study, failing to correlate the biohumoral data observed with the diet of the individuals analyzed.

On the other hand, the control group was matched for age and sex assigned at birth, making densitometric and body composition comparisons effective.

## Conclusions

In our study, AMAB transgender adults had lower bone density, lean mass and strength prior to GAHT. On the other hand, AFAB individuals had similar bone density, but more fat mass in comparison to cisgender women. Both AFAB and AMAB showed significantly lower vitamin D levels. Lifestyle factors, such as physical inactivity and tobacco consumption, may negatively influence body composition and bone development. Early interventions such as physical exercise which enhance the lean mass and the mechanical load on the bone during adolescence, adequate dietary calcium intake, vitamin D supplementation, and smoke cessation might be beneficial to improve general and bone health in transgender people. Further studies are needed to better define which risk factors affect bone development in the transgender population.

### Supplementary Information

Below is the link to the electronic supplementary material.

Supplementary file1 (DOCX 13 KB)
