# Supplementary material for: Bone health and body composition in transgender adults before gender-affirming hormonal therapy: data from the COMET study
Source: J Endocrinol Invest. 2023 Jul 14;47(2):401–10. doi: 10.1007/s40618-023-02156-7 (PMC10859333; doi:10.1007/s40618-023-02156-7)
Supplement: Supplementary file 1 — Supplementary file1 (DOCX 13 KB) [file 40618_2023_2156_MOESM1_ESM.docx]

**Supplementary Table 1. Hormonal profile in transgender people**

|  | **Assigned Females At Birth (AFAB)**  **n= 78** | **Assigned Males At Birth (AMAB)**  **n=47** |
| --- | --- | --- |
| Testosterone [ng/mL] | 0.38  (0.27;0.50) | 5.31  (3.72;6.83) |
| Estradiol [ng/L] | 52.50  (36.00;98.00) | 31.00  (24.50;34.39) |
| LH [UI/L] | 6.29  (4.60;8.35) | 4.70  (3.71;6.05) |
| FSH [UI/L] | 6.20  (4.10;8.12) | 4.30  (2.75;6.15) |

*Notes:* values are expressed as mean ± standard deviation.

*Abbreviations:* AFAB = Assigned Female At Birth; AMAB = Assigned Male At Birth; LH: Luteinizing hormone; FSH: Follicle-Stimulating Hormone.
